# Improved co-registration of ex-vivo and in-vivo cardiovascular magnetic resonance images using heart-specific flexible 3D printed acrylic scaffold combined with non-rigid registration

**DOI:** 10.1186/s12968-019-0574-z

**Published:** 2019-10-10

**Authors:** John Whitaker, Radhouene Neji, Nicholas Byrne, Esther Puyol-Antón, Rahul K. Mukherjee, Steven E. Williams, Henry Chubb, Louisa O’Neill, Orod Razeghi, Adam Connolly, Kawal Rhode, Steven Niederer, Andrew King, Cory Tschabrunn, Elad Anter, Reza Nezafat, Martin J. Bishop, Mark O’Neill, Reza Razavi, Sébastien Roujol

**Affiliations:** 1 School of Biomedical Engineering and Imaging Sciences, King’s College, London, Fourth Floor Lambeth Wing, St Thomas’ Hospital, London, SE1 7EH UK; 2 Siemens Healthcare Limited, Frimley, UK; 3 Medical Physics, Guy’s and St. Thomas’ NHS Foundation Trust, London, UK; 4 Cardiology Department, University of Pennsylvania, Philadelphia, PA USA; 5 Cardiology Department, Beth Israel Deaconess Medical Centre / Harvard Medical School, Boston, MA USA

**Keywords:** Ex-vivo CMR, Co-registration, 3D printing, Scar imaging

## Abstract

**Background:**

Ex-vivo cardiovascular magnetic resonance (CMR) imaging has played an important role in the validation of in-vivo CMR characterization of pathological processes. However, comparison between in-vivo and ex-vivo imaging remains challenging due to shape changes occurring between the two states, which may be non-uniform across the diseased heart. A novel two-step process to facilitate registration between ex-vivo and in-vivo CMR was developed and evaluated in a porcine model of chronic myocardial infarction (MI).

**Methods:**

Seven weeks after ischemia-reperfusion MI, 12 swine underwent in-vivo CMR imaging with late gadolinium enhancement followed by ex-vivo CMR 1 week later. Five animals comprised the control group, in which ex-vivo imaging was undertaken without any support in the LV cavity, 7 animals comprised the experimental group, in which a two-step registration optimization process was undertaken. The first step involved a heart specific flexible 3D printed scaffold generated from in-vivo CMR, which was used to maintain left ventricular (LV) shape during ex-vivo imaging. In the second step, a non-rigid co-registration algorithm was applied to align in-vivo and ex-vivo data. Tissue dimension changes between in-vivo and ex-vivo imaging were compared between the experimental and control group. In the experimental group, tissue compartment volumes and thickness were compared between in-vivo and ex-vivo data before and after non-rigid registration. The effectiveness of the alignment was assessed quantitatively using the DICE similarity coefficient.

**Results:**

LV cavity volume changed more in the control group (ratio of cavity volume between ex-vivo and in-vivo imaging in control and experimental group 0.14 vs 0.56, *p* < 0.0001) and there was a significantly greater change in the short axis dimensions in the control group (ratio of short axis dimensions in control and experimental group 0.38 vs 0.79, *p* < 0.001). In the experimental group, prior to non-rigid co-registration the LV cavity contracted isotropically in the ex-vivo condition by less than 20% in each dimension. There was a significant proportional change in tissue thickness in the healthy myocardium (change = 29 ± 21%), but not in dense scar (change = − 2 ± 2%, *p* = 0.034). Following the non-rigid co-registration step of the process, the DICE similarity coefficients for the myocardium, LV cavity and scar were 0.93 (±0.02), 0.89 (±0.01) and 0.77 (±0.07) respectively and the myocardial tissue and LV cavity volumes had a ratio of 1.03 and 1.00 respectively.

**Conclusions:**

The pattern of the morphological changes seen between the in-vivo and the ex-vivo LV differs between scar and healthy myocardium. A 3D printed flexible scaffold based on the in-vivo shape of the LV cavity is an effective strategy to minimize morphological changes in the ex-vivo LV. The subsequent non-rigid registration step further improved the co-registration and local comparison between in-vivo and ex-vivo data.

## Background

Pre-clinical imaging experiments offer the opportunity for the validation of in-vivo imaging findings against high-resolution ex-vivo imaging examination. Ex-vivo cardiovascular magnetic resonance (CMR) combines the strengths of CMR tissue characterization without the limitation of tissue movement that occurs in-vivo and with greater flexibility for duration of acquisition while maintaining tissue 3D structural integrity. Ex-vivo CMR can achieve image resolution and quality surpassing that available during in-vivo CMR [1, 2] and has established the ability of in-vivo CMR to accurately characterize pathological processes. For example, ex-vivo CMR demonstrated the ability of late gadolinium enhanced (LGE) CMR imaging to differentiate viable from non-viable tissue in the chronic stage after myocardial infarction (MI) [3], that gadolinium accumulation differentiates myocardial scar from adjacent healthy tissue at a near cellular level [2] and has been used to accurately establish the area at risk (AAR) following experimental MI [4].

**Fig. 1:**
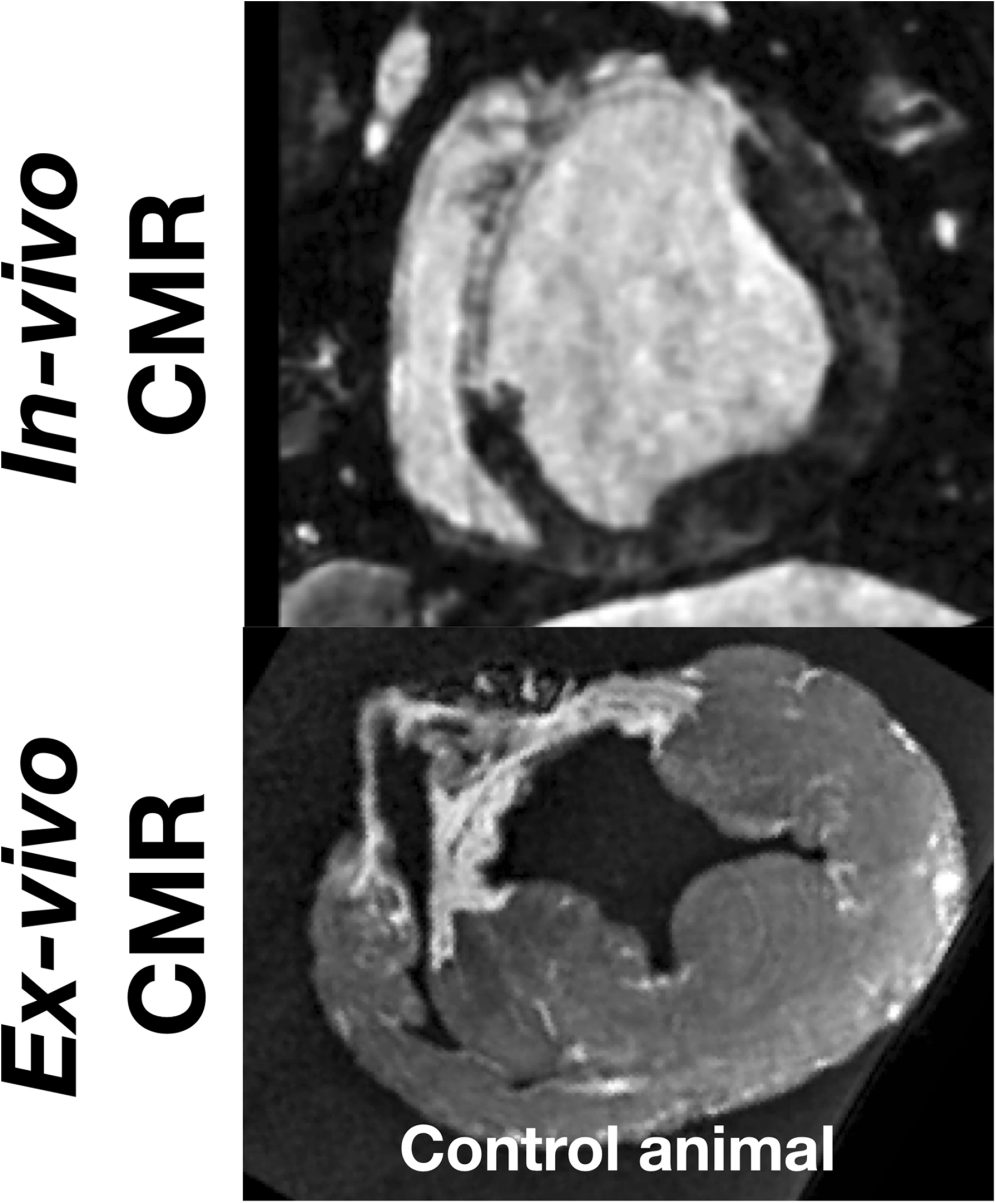
Preliminary data demonstrating motivation for study. Corresponding short axis view from in-vivo CMR (top panel) and ex-vivo CMR (bottom panel) in which no support for the left ventricular cavity was provided during ex-vivo imaging

Despite these advantages, relating ex-vivo imaging findings to in-vivo observations is still associated with significant challenges. Within the thorax, the position of the heart is maintained by the great vessels and enclosure within the pericardial sac. The shapes of the chamber cavities and myocardium are affected by the internal cavity pressure and intrathoracic pressure as well as passive and active tension within the myocardial wall. When the heart is removed from the thorax it is no longer subject to these forces, which can result in a significant change in the shape of the heart, imposing a barrier to accurate registration back to in-vivo imaging, as shown in Fig. 1. In addition, tissue volumes change on transition to the ex-vivo condition, even prior to fixation, with implications for comparisons of tissue volumes between in-vivo and ex-vivo imaging [5]. Ideally, the comparison of structural features between in-vivo and ex-vivo imaging requires the accurate registration of in-vivo and ex-vivo data, which may be considered in two stages: The first step is maintaining the shape of the heart in the ex-vivo condition as close to its shape in the in-vivo condition as possible despite the changes in loading conditions and myocardial tissue state. The second step is the subsequent registration of the in-vivo and ex-vivo imaging data for direct comparison.

Various techniques have been proposed to overcome the tendency of the left ventricle (LV) to collapse when removed from the thorax, including inflating balloons with deuterated water within the LV cavity [6, 7] and injecting a setting resin into the LV cavity [8] or the coronary arteries [9, 10] prior to ex-vivo assessment. While effective at preventing LV collapse these techniques do not necessarily preserve the in-vivo shape of the LV cavity.

3D printing is a widely available technology and shows promise for the personalized assessment and management of a range of medical conditions as well as research applications. In clinical practice, 3D printed models offer incremental benefit over contemporary imaging when used in the planning of thoracic surgical procedures, which may reflect more effective transmission of imaging information to operators. 3D printed models have been used for optimization of prosthetic heart valve size selection [11] and to produce anatomically customized implants and prostheses in trauma surgery [12]. Furthermore, 3D printed MRI derived mounts for tissue analysis improve registration of CMR imaging to ex-vivo tissue samples when compared with conventional techniques [5, 13], facilitating accurate interpretation of histological specimens.

In this study, we developed and assessed a novel two-stage approach to optimize the registration of in-vivo and ex-vivo CMR in a porcine model of chronic MI. A flexible 3D printed scaffold based on a direct segmentation of in-vivo imaging in the shape of the LV cavity and aortic root was employed with the goal of preserving the intra-thoracic shape of the LV during ex-vivo CMR. Subsequently, a method for non-rigid registration of the ex-vivo imaging to the in-vivo imaging was applied. A comparison of the chamber volumes and the correspondence between areas of myocardial scar between the in-vivo and ex-vivo data was undertaken after each step. The results were compared with a cohort of experiments performed without the use of a 3D printed insert to maintain LV shape during ex-vivo imaging.

## Methods

### Experimental myocardial infarction

The experimental protocol was approved by local and national institutional animal care and ethics committees and were performed at the Institute de Chirurgie Guidée par l’image (IHU), Strasbourg, France and the Beth Israel Deaconess Medical Centre, Boston, USA. Twelve domestic swine underwent a 180 min balloon occlusion of the mid left anterior descending artery to create experimental ischemia-reperfusion MI as previously described [14]. Seven animals formed the experimental group and five animals formed the control group.

### Experimental group

#### In-vivo imaging

All imaging was performed on a 1.5 T scanner (MAGNETOM Aera, Siemens Healthineers, Erlangen, Germany). Each pig underwent in-vivo LGE CMR at 6 weeks post MI. 15 min after 0.1 mmol/kg bolus of Gadovist (gadobutrol, Gd-DO3A-butrol; Bayer Healthcare, Berlin, Germany), Gadovist infusion (0.0011 mmol/kg/min) was commenced as previously described [15] and 15 min later LGE images were acquired (during continuous contrast administration) using an isotropic navigator-gated ECG-triggered 3D inversion recovery sequence with a balanced steady-state free precession readout (coronal orientation; linear k-space reordering; TE/TR/α = 1.58 ms/3.6 ms/90°; gating window = 7 mm; parallel imaging using GRAPPA with an acceleration factor of 2; voxel size = 1.2 × 1.2 × 1.2mm^3;^ Field of view (FOV) = 400 × 257 × 96 mm^3^, with full ventricular coverage).

#### Optimization of LV shape during ex-vivo imaging

The LV blood pool excluding papillary muscles and including aortic root was manually segmented from the in-vivo 3D LGE CMR using the open source Medical Imaging Toolkit (MITK, http://itk.org/) based CEMRG package (www.cemrg.com). The segmentation geometry was refined within 3-Matic Medical 10.0 (Materialise NV, Leuven, Belgium). The 3D scaffold was printed in a flexible material (TangoPlus FullCure930 plastic) using an Objet500 Connex1, polyjet 3D printer (Objet-Stratasys, Israel). The scaffold was printed with a spatially varying thickness of 6 mm for the borders of the LV blood pool and 4 mm for the aortic root to improve the compressibility of this portion. The disc at the mitral valve (MV) plane was printed with a thickness of 8 mm to improve the rigidity of this portion of the scaffold and a 6 mm hole was left in this disc to remove the support material required during the printing process.

#### Ex-vivo data acquisition

**Fig. 2:**
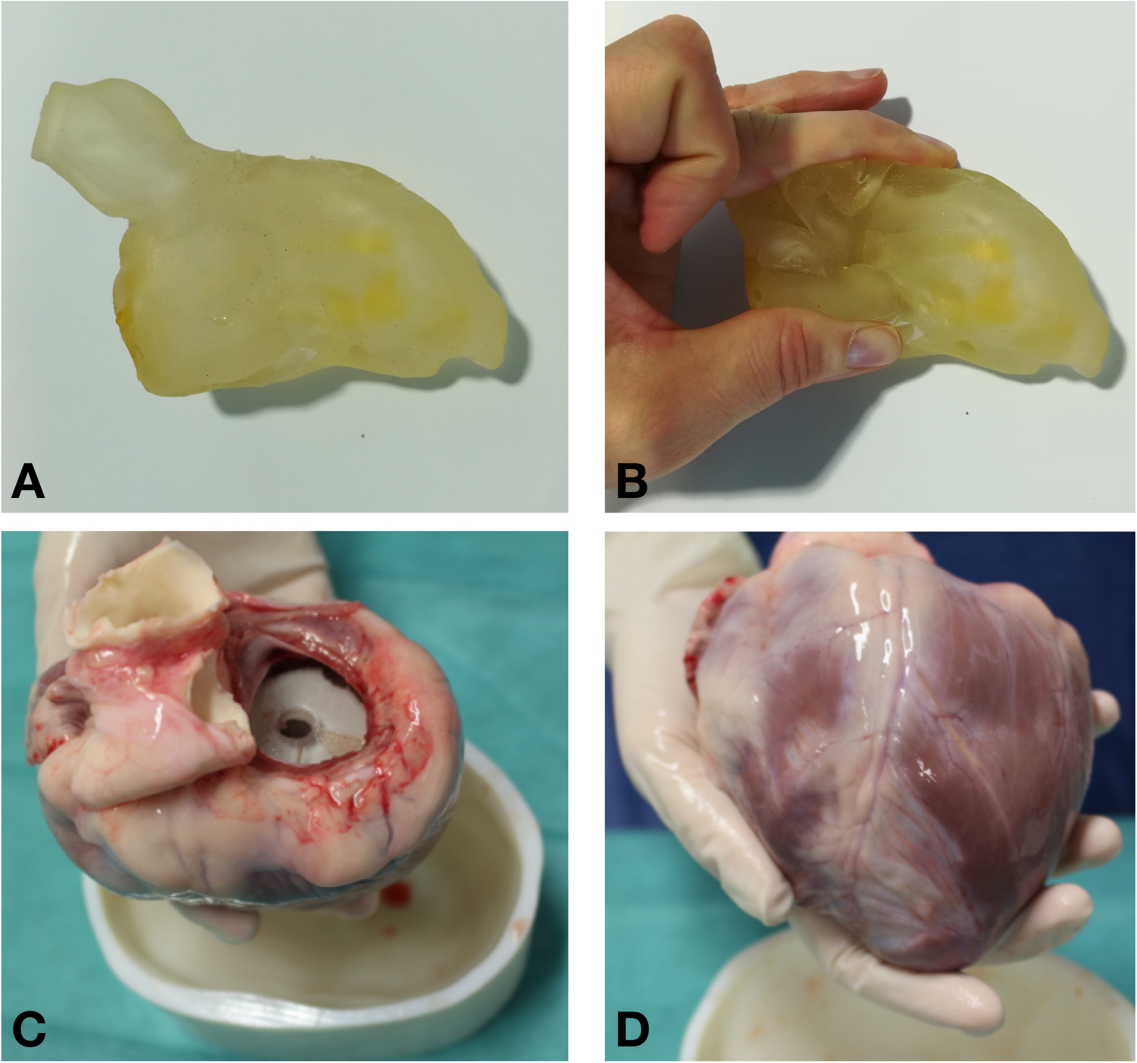
Panel **a** 3D printed scaffold. Panel **b** Flexibility of 3D printed scaffold allowed passage across the mitral valve (MV). Panel **c** Basal aspect of heart in which 3D printed is seen through the MV. Panel **d** Anterior view of heart containing scaffold

One week after in-vivo CMR imaging and 15 min prior to euthanasia a further dose of Gadovist (0.2 mmol/kg) was administered intravenously. The heart was arrested in diastole using intravenous potassium chloride (KCl). The thorax was opened, the great vessels cut, and the heart removed, preserving the aortic root. The hearts were rinsed and bathed in 0.9% saline and additional intra-coronary KCl administered. The heart-specific 3D printed flexible scaffold of the in-vivo LV blood pool and aorta was then inserted into the excised heart as follows: the 3D printed scaffold was manually compressed (as shown in Fig. 2) and then passed into the LV cavity across the MV. Correct orientation of the scaffold was ensured by positioning the aortic portion of the scaffold across the aortic valve. Hearts from all animals underwent ex-vivo imaging suspended in a bath of saline using sutures inserted into the mitral annulus. The saline submerged the heart completely during ex-vivo imaging, which was performed using an isotropic 3D T1-weighted spoiled gradient echo sequence with the following parameters: TE/TR/α = 11.2 ms/5.41 ms/20°; bandwidth = 130 Hz/Px; voxel size = 0.4 × 0.4 × 0.4 mm^3^; FOV = 150 × 150 × 100 mm^3^, number of averages = 3. Imaging commenced within an hour of removing the heart. Total acquisition time was approximately 3 h reflecting the time course over which the distribution of Gadovist was expected to remain stable [2].

#### Segmentation

Each series of in-vivo and ex-vivo images were manually segmented in Seg3D2, an Insight Segmentation and Registration Toolkit (ITK, https://itk.org/) based tool, by a single observer. On the in-vivo imaging papillary muscles were included in the LV cavity segmentation if there was contrast between the papillary muscle body and the endocardial surface, and otherwise they were included in the LV myocardial segmentation. On the ex-vivo imaging, papillary muscles were also included in the LV cavity segmentation when a rim of contrast was evident between the muscle body and the LV wall, in order to consistently identify papillary muscle that would have been included in the LV cavity segmentation on the in-vivo imaging, despite the fact that in the ex-vivo condition it was pushed against the LV wall by the scaffold (illustrated in Additional file 1). The in-vivo and the ex-vivo segmentations were terminated at a circular disc in the MV plane. On the in-vivo imaging, scar was segmented according to a full-width at half-maximum (FWHM) strategy [16] followed by a connected component filter with seeds placed in clearly enhancing regions of myocardium within the vascular territory of the infarct.

On the ex-vivo imaging, scar was segmented as follows. A non-enhancing region of interest (ROI) within the basal myocardial segmentation, remote from the vascular territory of the infarct, was segmented. In addition, an ROI in a region of increased SI within the vascular territory of the infarct was manually segmented. The SI histograms from these ROIs were exported and saved in Matlab format. The lower threshold for scar was automatically calculated within Matlab by setting the lower SI boundary of scar (SI_scar_) at one standard deviation below the mean SI within the scar ROI. Within Seg3D2, the threshold filter was used to select voxels with a SI above SI_scar_. A connected component filter with seeds placed in clear regions of scar was applied. Additional manual deletion of voxels that were deemed to be enhanced but remote from scar was performed. No voxels were added to the scar segmentation. The difference in image contrast between the ex-vivo and in-vivo imaging (as shown in Additional file 1: Figures S2 and S3) necessitated the use of different scar segmentation algorithms between the two imaging sets.

#### Co-registration of in-vivo to ex-vivo imaging

Two approaches were compared for the co-registration of ex-vivo to in-vivo datasets: Firstly, using rigid landmark-based co-registration only and secondly using non-rigid image co-registration.

Rigid landmark based registration [17, 18] of the in-vivo and original ex-vivo meshes was carried out within a custom written Matlab (Mathworks, Natick, Massachusetts, USA) based image analysis package (MedIACare) by operators blinded to the position of the scar on each mesh, using the same readily identifiable anatomical landmarks on the in-vivo and ex-vivo imaging. Specifically the landmarks chosen were the left main coronary artery at the point of bifurcation into the left anterior descending artery and the left circumflex artery, the right coronary artery ostium, the LV apex and papillary muscles at the point of insertion into the LV when this was unambiguously visualised.

Non-rigid image-based co-registration was performed on manually segmented binary data. Segmentations and images were exported from Matlab to the Neuroimaging Informatics Technology Initiative (NIfTI) format. The ex-vivo segmentations were downsampled to match the resolution of the in-vivo segmentations. In-vivo and downsampled ex-vivo segmentations were imported into RView, a Medical Image Registration Toolkit (MIRTK, http://biomedia.doc.ic.ac.uk/software/mirtk/) based tool, for visualisation and selection of landmarks [19]. Landmarks were selected on the in-vivo segmentation and ex-vivo segmentations corresponding to the identical anatomical landmarks chosen for the mesh-based registration. Following an initial landmark based registration, segmentations underwent automatic rigid, followed by affine and then non-rigid registration [20] using the Image Registration Toolkit (MIRTK) library [20] in a process in a process that has previously demonstrated excellent accuracy [21].

### Data analysis

#### Native multi-modal comparison

LV dimensions were assessed on in-vivo and ex-vivo imaging by measuring the LV vertical long axis (apex to the MV annular plane) dimension and two approximately perpendicular measurements of the cavity diameter in basal short axis views. LV cavity and myocardium volumes were calculated by manual segmentation of the endocardial and epicardial border of the myocardium. Scar volumes were calculated by signal intensity thresholding of the manually segmented myocardium. All tissue volumes were calculated from binary segmentations after downsampling of the ex-vivo data to match the in-vivo data resolution. Endocardial and epicardial meshes were automatically generated from these binary segmentations using MedIACare. Scar was defined on the endocardial mesh as those nodes in which a projection from the nearest epicardial surface node passed through a minimum of one voxel on the segmentation assigned as scar. Wall thickness at the corresponding location was assessed as the absolute distance between these nodes. Scar transmurality was calculated as the proportion of the projection that was within voxels labelled as scar.

#### Anatomical comparison following co-registration

Nodal-wise DICE coefficient for scar between the in-vivo and registered ex-vivo endocardial meshes was calculated. To this end, a projection was made from the endocardial surface of the in-vivo mesh onto the endocardial surface of the registered ex-vivo meshes [22]. Voxel-wise DICE coefficient was calculated for both LV cavity and myocardium between the in-vivo and the non-rigidly registered ex-vivo segmentations [22]. LV cavity volume, myocardial volume and wall thickness were also evaluated for the non-rigidly registered ex-vivo data using the same strategy as described in the previous section.

### Control group

Five animals underwent in-vivo and ex-vivo imaging with similar parameters (details in Additional file 1). No printed scaffold was used to maintain the LV cavity shape during ex-vivo imaging in the control group. LV dimensions, LV cavity and myocardial volume were assessed from the in-vivo and ex-vivo imaging using the same method as for the experimental group.

### Statistical analysis

Normality of distribution of variables was assessed using the Shapiro Wilk’s test. Normally distributed continuous variables are expressed as mean ± standard deviation (SD). Normally distributed parameters were compared with two tailed paired samples *t*-test or one-way ANOVA with Tukey post hoc analysis where indicated. Scar volumes in the in-vivo and ex-vivo state were compared using simple linear regression. A result was considered statistically significant at the 5% significance level (*p* < 0.05). Statistical analysis was carried out in SPSS (v24, IBM Corporation, New York).

## Results

Segmentation of the in-vivo imaging for preparation of the scaffold took less than 10 minutes in each case. Refinement of the segmentation prior to printing was completed in each case in under 30 min and the printing was then completed within 180 min, followed by a further 30 min of post processing in each case. Ex-vivo imaging using a 3D scaffold was successfully acquired in all swine. An example of a 3D printed scaffold inserted into an excised heart is shown in Fig. 2. In 6 cases the scaffold was successfully passed across the MV. In a single case an incision at the basal infero-lateral wall was required because the MV annulus was too small to allow passage of the compressed scaffold. External examination demonstrated that the scaffold maintained the LV shape during ex-vivo CMR acquired over 4 h and there was low signal from the scaffold, which made identification of the endocardium unambiguous.

**Fig. 3:**
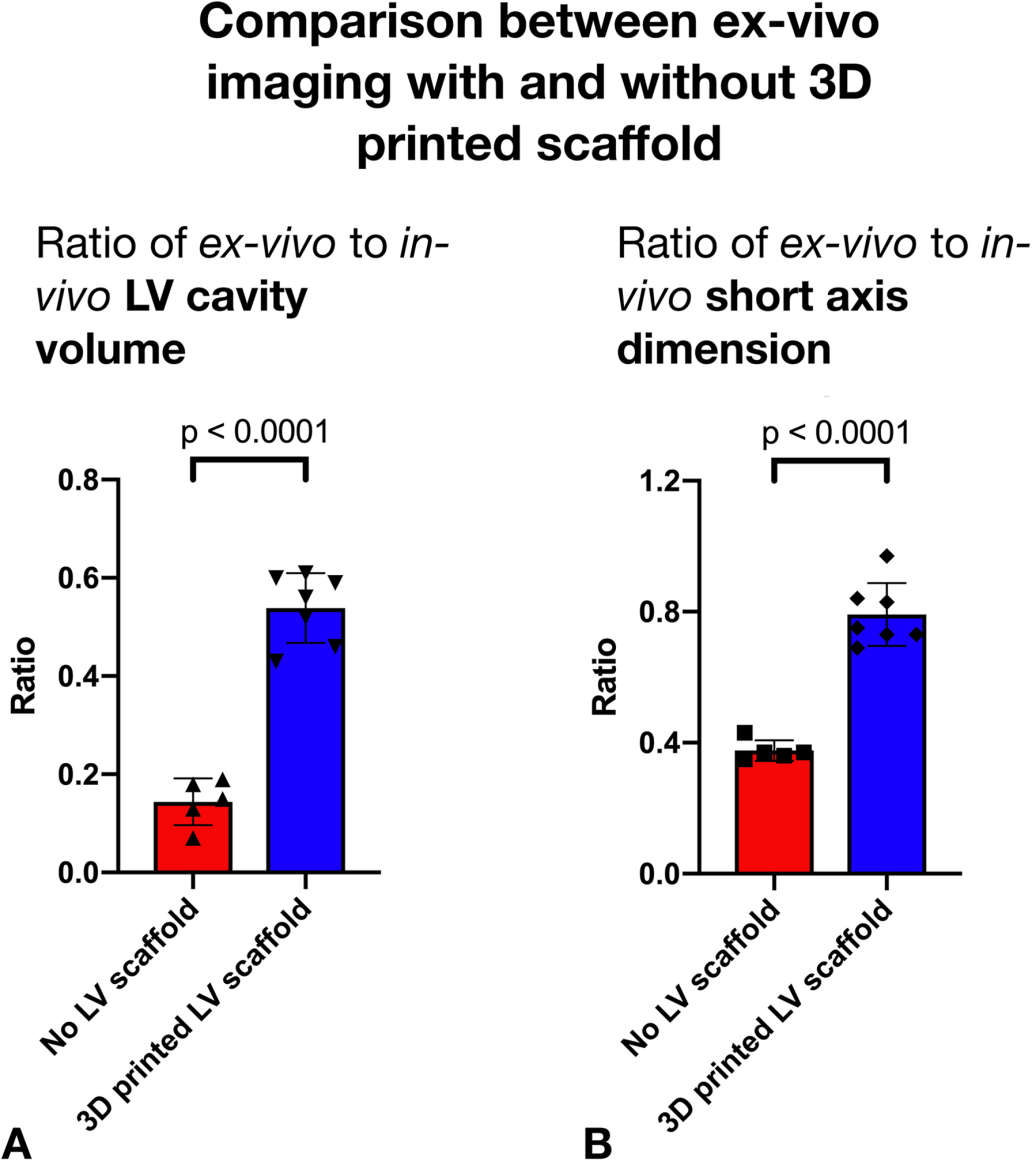
Panel **a** Comparison of ratio of in-vivo to ex-vivo LV cavity volume between control and experimental group. Panel **b** Comparison of ratio of in-vivo to ex-vivo short axis dimension between control and experimental group

Myocardium was readily segmented from each of the ex-vivo scans and a region of increased signal intensity in the antero-septal wall was clearly visualized (example in Additional file 1: Figure S1). An example of corresponding positions within the myocardium on in-vivo imaging and ex-vivo imaging is shown in Fig. 3.

### Native multi-modal comparison

#### Control group

Comparison of the LV dimensions between the in-vivo and ex-vivo imaging demonstrated a mean ratio of 0.82 in the long axis (95% CI 0.77–0.87, *p* = 0.0034) and 0.38 in the short axis (95% CI 0.35–0.41, *p* < 0.001). The segmented ex-vivo LV cavity volume (mean volume = 17 ml) had a mean ratio of 0.14 (95% CI 0.10–0.19, *p* < 0.001) to the in-vivo LV cavity volume (mean volume = 130 ml). The volume of the segmented ex-vivo myocardial tissue volume (mean = 118 ml) had a mean ratio of 1.36 (95% CI 1.11–1.60, *p* = 0.0081) to the in-vivo myocardial tissue volume (mean = 114 ml) (Fig. 3).

#### Experimental group

**Fig. 4:**
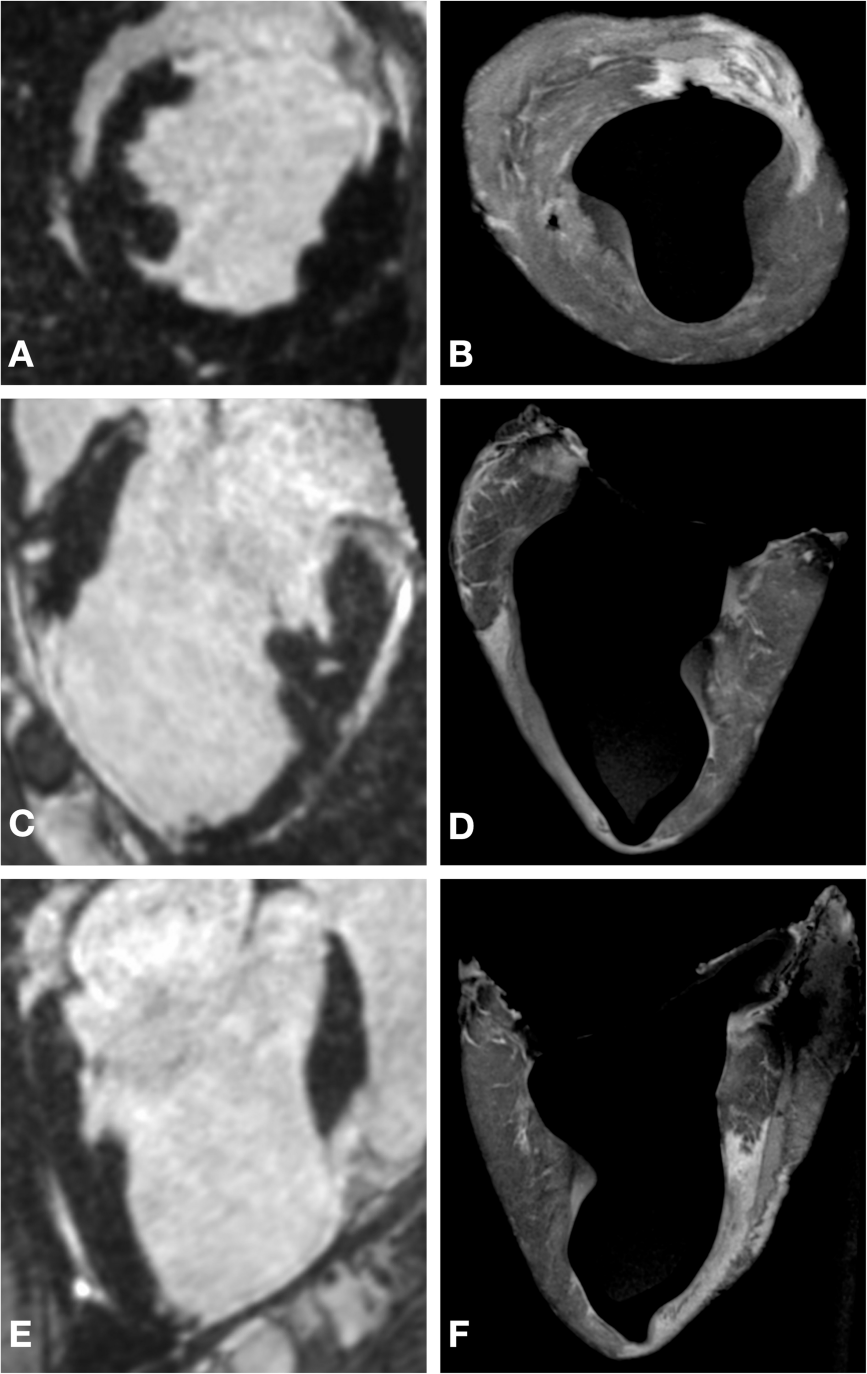
Panel **a** and **b** Multiplanar reconstruction (MPR) in short axis orientation from in-vivo (**a**) and ex-vivo (**b**) imaging. Panel **c** to **f** MPR in 2-chamber (**c** and **d**) and three chamber (**e** and **f**) orientations from in-vivo and ex-vivo imaging

Comparison of the LV dimensions between the in-vivo and ex-vivo imaging (example shown in Fig. 4) demonstrated a mean ratio of 0.83 in the long axis (95% CI 0.78–0.88, *p* < 0.001) and 0.81 in the short axis (95% CI 0.76–0.86, p = < 0.001). The segmented ex-vivo LV cavity volume (mean volume = 79 ml) had a mean ratio of 0.56 (95% CI 0.47–0.66, *p* < 0.001) to the in-vivo LV cavity volume (mean volume = 143 ml). The volume of the segmented ex-vivo myocardial tissue volume (mean = 150 ml) had a mean ratio of 1.29 (95% CI 1.16–1.45, *p* = 0.0013) to the in-vivo myocardial tissue volume (mean = 114 ml).

#### Comparison of dimension changes in experimental and control groups

When the LV cavity volume ratio between ex-vivo and in-vivo imaging was compared between the control group and the 3D printed insert group there was a significantly greater change in cavity volume in the control group (ratio of cavity volume between ex-vivo and in-vivo imaging in control and experimental group 0.14 vs 0.56, *p* < 0.001). Comparison of the short and long axis dimensions between ex-vivo and in-vivo imaging demonstrated that there was a significantly greater change in the short axis dimensions in the control group (ratio of short axis dimensions in control and experimental group 0.38 vs 0.79, *p* < 0.001) (Fig. 3) while the ratio of long axis dimensions on ex-vivo and in-vivo imaging between control and experimental group was not significantly different (0.82 v 0.83, *p* = 0.74). The ratio of myocardial volume on ex-vivo and in-vivo imaging between the control and experimental group was not significantly different (ratio 1.29 vs 1.36, *p* = 0.51).

### Experimental group scar analysis

**Fig. 5:**
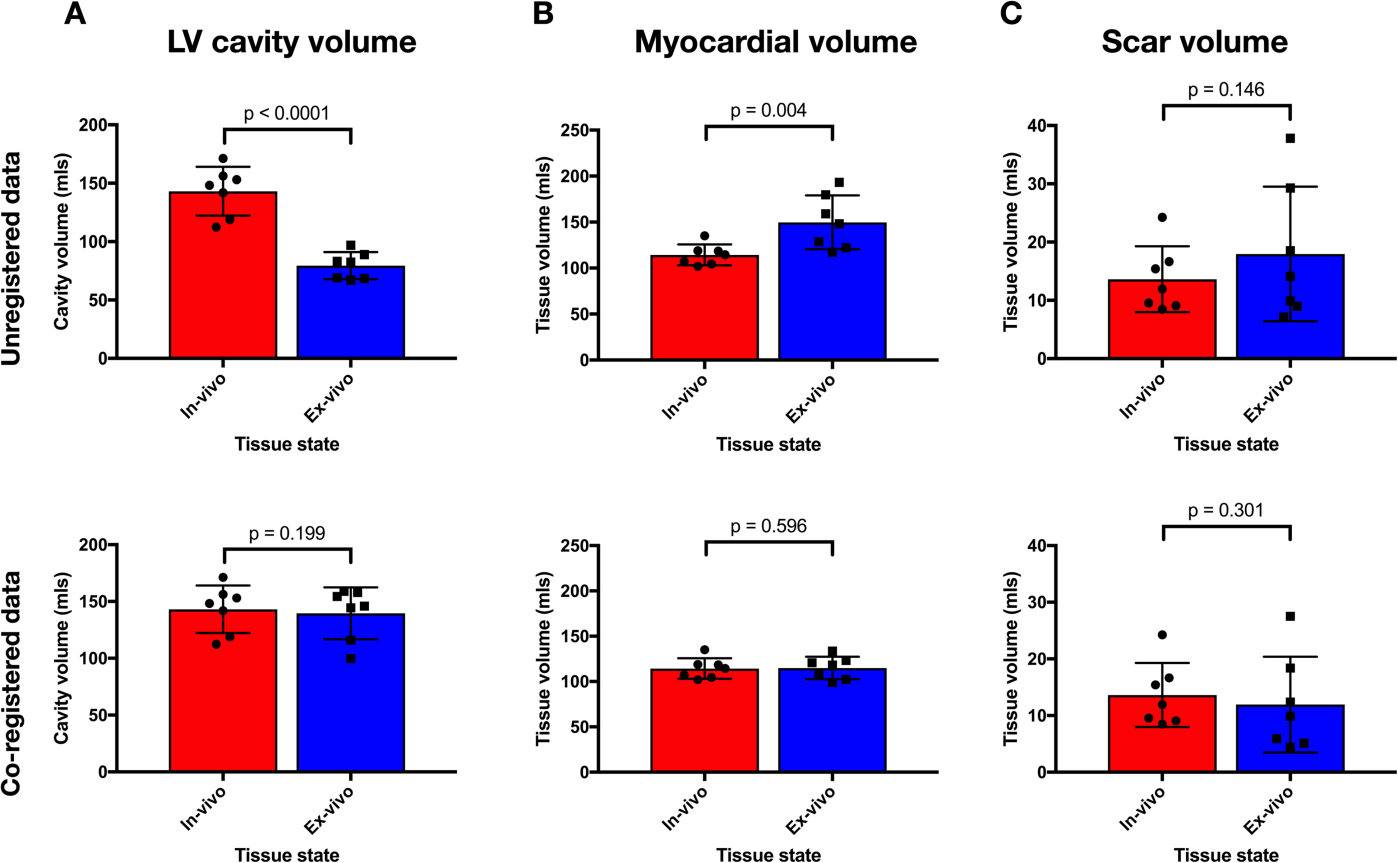
Comparison of tissue volumes between the in-vivo and ex-vivo state. Bars indicate mean volume of LV cavity (column **a**), myocardial tissue (column **b**) and scar (column **c**) segmented from in-vivo imaging (red) and ex-vivo imaging (blue), before (top row) and after (bottom row) completer two-step co-registration process. Individual data points are represented by circles (in-vivo) and squares (ex-vivo) and error bars show standard deviation. *P* values for ratio paired *t-test* are shown

In the experimental group, the mean volume of scar was greater in the ex-vivo condition (mean = 18.0 ml) than in the in-vivo condition (mean volume = 13.6 ml) but the difference did not reach statistical significance (*p* = 0.15) (Fig. 5). There was a good correlation between volume of scar in the in-vivo and ex-vivo condition after the resampling of the ex-vivo data (see Additional file 1). Scar volume as a proportion of total LV myocardium in the in-vivo imaging was 11.9 ± 4.6% compared to 11.4 ± 5.9% in the ex-vivo imaging (*p* = 0.73). There was a significant increase in mean tissue thickness between the in-vivo and ex-vivo imaging in healthy regions, from 9.95 ± 2.78 mm to 12.71 ± 3.48 mm (*p* = 0.010). There were no statistically significant differences in tissue thickness between in-vivo and ex-vivo imaging in regions of 0–50% scar transmurality (9.28 ± 2.54 mm vs. 9.65 ± 3.84 mm, *p* = 0.64), or regions of 50–100% scar transmurality (7.59 ± 1.94 mm vs. 7.40 ± 2.07 mm, *p* = 0.72). Proportional change in tissue thickness was statistically significantly different between healthy tissue, regions of 0–50% scar transmurality and regions of 50–100% scar transmurality, F [2, 18] = 4.146, *p* = 0.033. Proportional change in tissue thickness was greatest in the healthy tissue (mean change in thickness 29 ± 21%), then the regions of 0–50% scar transmurality (mean change 5 ± 4%) and then the regions of 50–100% scar transmurality (mean change − 2 ± 2%). Tukey post hoc analysis revealed that only the difference in proportional change in tissue thickness between regions of 50–100% scar transmurality and healthy tissue was statistically significant (30, 95%CI 2 to 59%, *p* = 0.034).

### Anatomical comparison following co-registration

The segmentation-derived meshes of the in-vivo imaging and the ex-vivo imaging were compared following mesh-based landmark registration and image-based non-rigid registration.

**Fig. 6:**
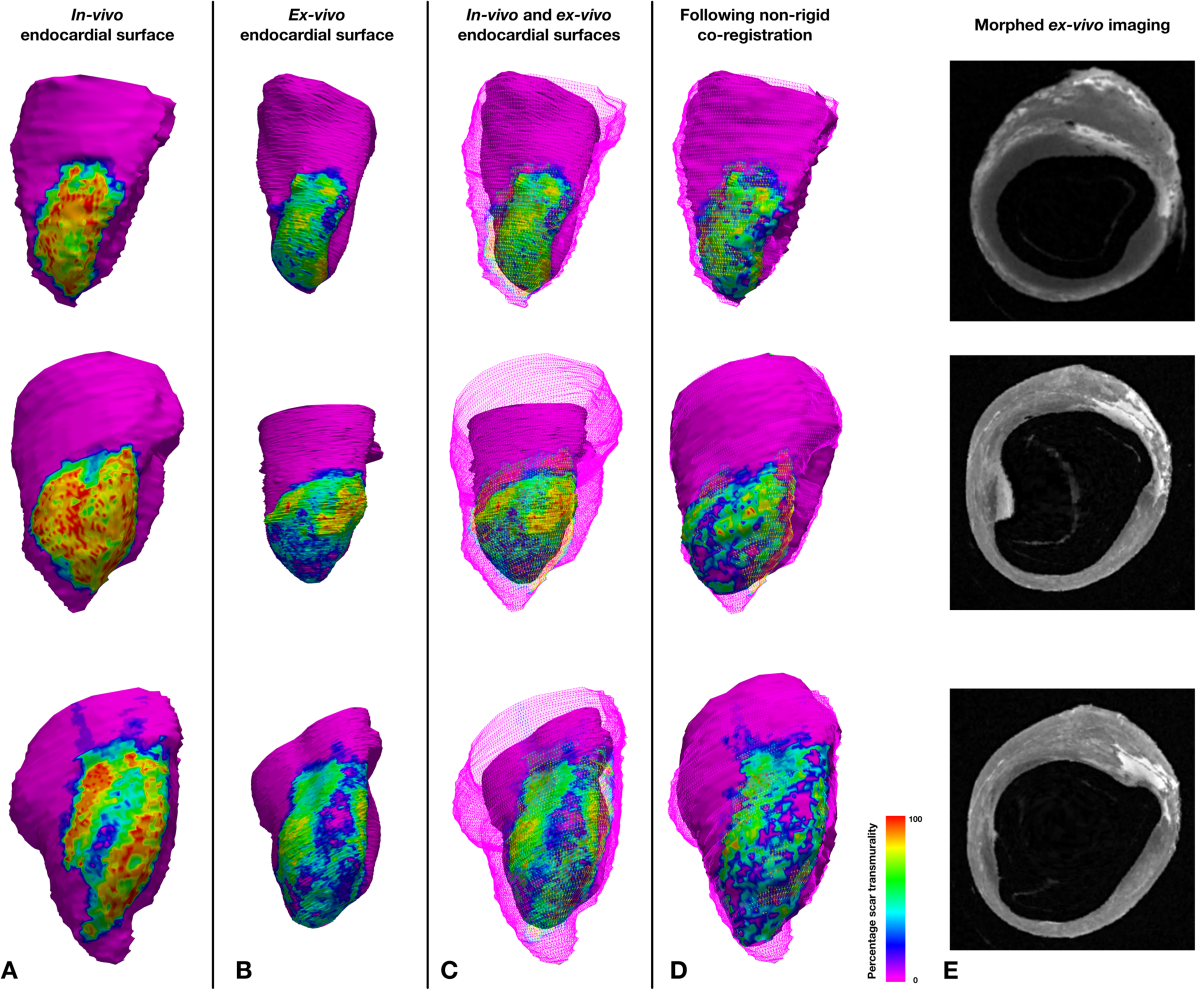
Examples of rigid and non-rigid co-registration of in-vivo and ex-vivo data. Each row contains data from an individual case. Column **a** Mesh of in-vivo endocardial surface color coded according to projected scar transmurality. Column **b** Mesh of co-registered ex-vivo endocardial surface color coded as per in-vivo meshes. Column **c** Wire frame representation of in-vivo endocardial mesh and surface of co-registered ex-vivo endocardial mesh. Column **d** Wire frame representation of in-vivo endocardial mesh and surface of co-registered ex-vivo endocardial mesh following non-rigid co-registration. Column **e** Morphed ex-vivo imaging following non-rigid registration of the examples shown

Following landmark-based registration of the extracted meshes, the ex-vivo endocardial surface was smaller than the in-vivo endocardial surface, reflecting LV cavity shrinkage in the ex-vivo condition*.* Despite the smaller shape of the ex-vivo mesh, visually there was good correspondence between the shape of the endocardial surface and the region of scar (Figs. 5 and 6). The mean DICE similarity coefficient for scar projected from the epicardial to endocardial surfaces was 0.75 (±0.03).

**Fig. 7:**
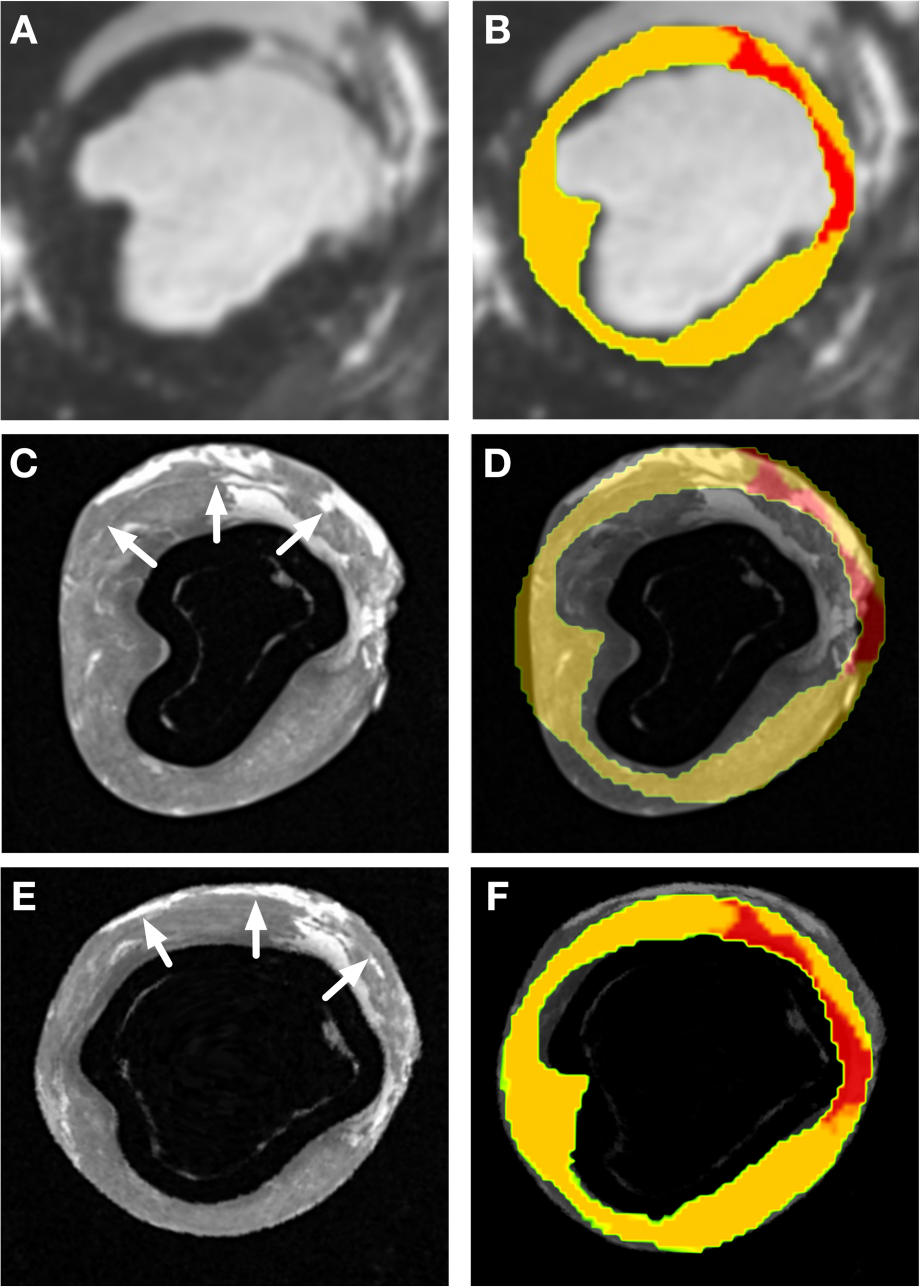
Panel **a** Mid ventricular short axis slice from in-vivo imaging showing antero-septal scar. Panel **b** Short axis slice at same level as panel A with segmentation of healthy myocardium (yellow) and scar (red) superimposed on imaging. Panel **c** Mid ventricular short axis slice from ex-vivo imaging at corresponding level to panel A/B. White arrows indicate the epicardial aspect of the interventricular septum, with the adjacent collapsed right ventricular cavity. Panel **d** Short axis slice at same level as panel C with segmentation of in-vivo imaging superimposed. Note is made of the shrinkage of the left ventricular cavity despite the printed scaffold. Panel **e** Mid ventricular short axis slice from ex-vivo imaging at corresponding level to panel C/D following non-rigid registration to correct for residual morphological change. White arrows indicate the epicardial aspect of the interventricular septum, with the adjacent collapsed right ventricular cavity. Panel **f** Short axis slice at same level as panel E with segmentation of in-vivo imaging superimposed demonstrating correction of residual morphological change following non-rigid registration

**Fig. 8:**
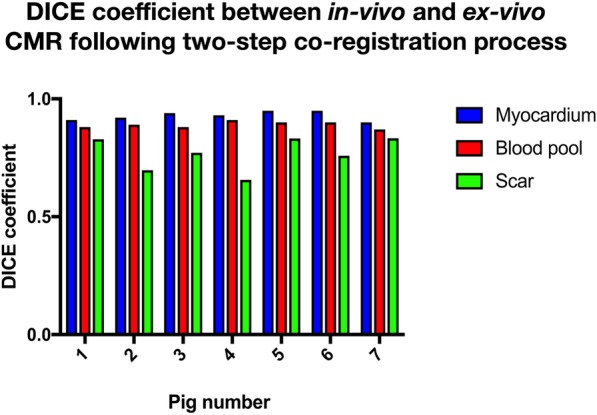
Shape comparison between in-vivo and ex-vivo imaging following non-rigid registration. DICE similarity co-efficient between myocardium, LV cavity and scar following non-rigid registration in 7 animals

Following image-based non-rigid registration of the ex-vivo segmentations, the myocardial and scar masks from the in-vivo and ex-vivo imaging appeared subjectively well aligned and the scar masks similar. A representative example of a short axis section from in-vivo imaging, the segmentation at this level and the corresponding ex-vivo imaging before and after non-rigid registration are shown in Fig. 7. The mean DICE similarity coefficient for myocardium, LV cavity, and scar are displayed in Fig. 8.

The DICE similarity coefficient calculated for the correspondence between regions of scar in the in-vivo and rigidly registered ex-vivo data demonstrate that the region of scar is well registered between the in-vivo and ex-vivo data sets following rigid registration alone (DICE coefficient = 0.75). Following non-rigid registration, the DICE coefficient for the scar rose to 0.77 which did not represent a statistically significant improvement (*p* = 0.55).

The mean DICE similarity coefficient for the myocardial mask segmentation and LV cavity were 0.93 (±0.02) and 0.89 (±0.01), respectively. The mean ratio of the volume of the segmented LV cavity on the in-vivo segmentations to the non-rigidly registered ex-vivo segmentation was 1.03 (mean volume in-vivo 143 ± 21 ml vs 140 ± 23 ml ex-vivo, *p* = 0.199) and the mean ratio of the volume of segmented myocardium between the in-vivo and ex-vivo condition was 1.00 (mean volume in-vivo 114 ± 11 ml vs 115 ± 12 ml ex-vivo, *p* = 0.60).

## Discussion

In this study, a two-stage co-registration process combining the use of a flexible, 3D printed scaffold and a non-rigid registration technique has been proposed and assessed to facilitate the accurate comparison between ex-vivo and in-vivo imaging. The main findings of this study are 1) the demonstration of non-uniform changes in tissue geometry that occur in the unloaded, ex-vivo condition and their quantification 2) the feasibility of using a flexible, 3D printed scaffold based on the in-vivo LV and aorta shape to prevent LV cavity collapse during ex-vivo scanning reducing LV shrinkage below 20% in each dimension and resulting in isotropic contraction of the LV cavity and myocardium, and 3) the demonstration of a subsequent non-rigid registration step to successfully correct for residual non-uniform changes in tissue geometry.

LV cavity shrinkage and wall thickening is clearly demonstrated in previous reports using ex-vivo CMR scanning [8, 23, 24], although comparisons of absolute volumes between the imaging techniques are infrequently reported. Tissue shape change in the ex-vivo state is the result of the removal of tethering structures, changes in loading conditions and muscle contraction due to the depletion of intracellular adenosine triphosphate (ATP), followed by an increase in the proportion of myocytes in the contracted state, prior to the onset of rigor mortis [25]. The proposed two stage approach enabled to fully correct for morphological changes occurring between in-vivo and ex-vivo imaging. Importantly, this framework is readily translatable to other in-vivo *and* ex-vivo imaging modalities [26, 27].

Optimizing the shape of the heart in the ex-vivo condition to best reflect the shape of the heart in the in-vivo condition was the first stage of this co-registration process. This step is key to ensure accurate non-rigid registration, which is more effective when initial geometric differences between the imaging data are minimised [28]. Data from the control experiments clearly demonstrated LV cavity collapse during ex-vivo imaging when no cavity support was used and the anisotropic contraction in this condition represents a significant obstacle which will reduce the accuracy of subsequent registration steps. Although in this experiment there was a delay of 1 week between in-vivo and ex-vivo data acquisition, the length of preparation of the scaffold would allow imaging to be acquired on the same day if required. An important consideration that informed the decision to use a flexible 3D printed scaffold was the ease with which the scaffold could be passed across the MV into the LV cavity. During preliminary experiments utilizing a rigid scaffold of the LV cavity [29], the maximum diameter of the LV occurred at a mid-ventricular level, and therefore in order to pass the scaffold across the MV annulus an incision was required in the MV annulus, as demonstrated in Additional file 1: Figure S2. In addition, due to the pseudo-symmetry of the LV cavity, the correct rotation of the scaffold around the LV long axis was uncertain. These experiences motivated the trial of a flexible 3D printed scaffold as assessed in this study. In all but one case using a flexible scaffold facilitated maintenance of LV cavity shape without incurring the disruption in tissue architecture that was required when using a rigid scaffold. The uncertainty of the rotation of the flexible scaffold was minimized by inclusion of the aortic root in the LV cavity segmentation, which was used a specific landmark to identify the correct rotation around the LV long axis, representing a significant benefit of using a flexible over a rigid 3D scaffold.

The ease with which the scaffold could be introduced into the LV cavity is dependent on its flexibility, which, for a given material, is influenced by the thickness with which the scaffold is printed. The printing thickness was subjectively optimized in prior experiments to achieve a balance between a scaffold that was stiff enough to avoid collapse due to LV shrinkage while flexible enough to be passed across the MV without the need to create an incision. While printing a thinner scaffold may have made it easier to pass across the MV annulus, it is likely that it would have been more susceptible to deformation when the tissue contracted ex-vivo*.* The most effective thickness to print such a flexible scaffold, or alternative strategies to prevent the collapse of a thinner scaffold, such as pressurising the cavity of the scaffold after insertion (for example using a balloon) or using a rigid scaffold that could be separated into parts of sufficiently small size to cross the MV annulus and be reconstructed inside the LV cavity, represent potential avenues for subsequent work.

When specific tissue compartments such as scar or AAR are compared between the ex-vivo and in-vivo condition they are commonly reported as a percentage of LV myocardium and good agreement between in-vivo and the ex-vivo proportions are reported [6, 7]. Our data highlight that although the proportion of myocardium defined as scar prior to registration is consistent, there is less tissue thickening in the scar than the remote healthy myocardium, indicating that tissue shape change is non-uniform when the infarcted heart is removed from the thorax. In-vivo CMR imaging was acquired at end diastole, at which point active tension within the myocardium is minimal and the LV end-diastolic pressure (LVEDP) is approximately 10 mmHg [30]. The differential response to tissue unloading between healthy tissue and fibrous scar observed may be explained by differences in stiffness between scarred and healthy myocardium [31], and likely also reflects more pronounced myocardial contraction in regions of healthy tissue. These non-uniform tissue volume changes, if uncorrected, further compound the difficulty of comparison between in-vivo and ex-vivo data [32, 33]. The co-registration framework presented maintains LV structural integrity, minimizes shape change during ex-vivo data acquisition and fully compensates for residual non-uniform shape changes.

The volume differences of the LV cavity, LV myocardium and scar between the ex-vivo and in-vivo imaging despite the use of a scaffold reflect the morphological changes between the two conditions that represent a residual limitation to their accurate comparison, motivating the addition of the second non-rigid registration step in the co-registration process. The close correspondence of the compartment volumes following this step, as shown in Fig. 5 indicate accurate compensation of the volume changes between ex-vivo and in-vivo conditions. The DICE similarity coefficient calculated for the correspondence between regions of scar in the in-vivo and rigidly registered ex-vivo data demonstrate that the region of scar is well registered between the in-vivo and ex-vivo data sets following rigid registration alone (DICE coefficient = 0.75). Following non-rigid registration, the DICE coefficient for the scar rose to 0.77 which did not represent a statistically significant improvement (*p* = 0.550). This reflects the success of the strategy to maintain the shape of the LV using the 3D printed scaffold as well as the reduced morphological changes evident in the scar tissue versus the more compliant neighboring healthy myocardium. While successful in matching the location of the scar subjectively and with a reasonable DICE similarity coefficient, the DICE of the scar (calculated nodal-wise) was lower than that achieved with the myocardium and the LV cavity (calculated voxel-wise). The scale dependence of the DICE similarity coefficient [34, 35] with the smaller relative volume of scar, different signal thresholding strategies employed for scar segmentation in both the in-vivo and ex-vivo imaging, and the lower resolution of the in-vivo imaging which is limited to represent complex patterns of scar demonstrated on high resolution ex-vivo imaging may contribute to this. The use of more advanced rigid co-registration for improved pre-conditioning [36] or other non-rigid co-registration approaches [37] could also be investigated to further improve scar matching. The increase in absolute tissue volume in the ex-vivo condition remains unexplained. The most likely explanation is absorption of saline into the tissue following submersion during imaging. It is uncertain if the additional volume is due to expansion of myocytes due to disruption of the cellular membrane or additional fluid in the extracellular space and a histological assessment would be required to establish this.

Data from ex-vivo CMR has played a crucial role in the validation of in-vivo CMR imaging, facilitating its widespread adoption as a tool for assessing the accuracy of in-vivo structural and functional myocardial imaging [1, 2, 38]. There is an expanding role for ex-vivo CMR in the investigation of the local structural basis for observed physiological phenomena, where it has been used to establish thresholds for the interpretation of in-vivo electrogram voltage data [23], which contribute to the identification of appropriate ablation targets during ventricular tachycardia (VT) ablation [39]. Ex-vivo CMR is the principle modality used to generate high resolution biophysical models. These models have been used to predict successful ablation targets for the treatment of post-MI VT [40] and been proposed as a basis for interpolating clinical resolution imaging to generate higher resolution estimations of local scar architecture [41], with the goal of translating the insight from biophysical models to clinical ablation procedures [42]. The accuracy of these results depend on matching the global 3D structure of the ventricle in regions of healthy myocardium as well as scar. The proposed co-registration strategy was successful in matching the volume and shape of the LV cavity, myocardium and scar between in-vivo and ex-vivo data and may thus provide a novel avenue to improve the accuracy of these results with potential significant pre-clinical and clinical impact.

This study has several limitations. In the in-vivo condition the papillary muscles are within the LV blood pool, while in the ex-vivo condition they are pushed against the LV endocardial wall by the printed insert, because the segmentation method used for the printed insert does not account for portion of the blood pool between the papillary muscle and the endocardial LV surface in-vivo*.* This is likely to have introduced a discrepancy between the LV cavity shape in the in-vivo and ex-vivo conditions. We believe the impact of this would have been small due to the proximity of the papillary muscles to the endocardial surface in-vivo and note that there was not a clear indication of more pronounced shape changes in this region. A more complex in-vivo segmentation process for design of the printed insert could be considered in future to address this limitation of the study. The study involved comparing the LV morphology between the in-vivo and ex-vivo condition, however different imaging sequences were used for each. This permitted acquisition of higher resolution ex-vivo imaging data but introduced the confounding effect of differences in the imaging sequences to the assessment of registration. The impact of the different imaging sequences on the blood pool and myocardial segmentation should be minimal as these are unambiguously defined in both data sets. Furthermore, prior to registration the ex-vivo imaging was resampled to the resolution of the in-vivo imaging data to minimize the impact of differences in image resolution on the comparison between the data sets. The impact of the differences in imaging sequence on scar segmentation is more challenging to establish. In addition, no consensus exists for the best strategy to threshold ex-vivo CMR images for the identification of scar. A simple and objective strategy for scar thresholding in the ex-vivo condition was selected to maintain consistency but may have contributed to small differences in scar assessment between the in-vivo to ex-vivo conditions. The correlation between scar volume between the in-vivo and ex-vivo data indicates that this resulted in relatively minor differences between tissue identified as scar between the two data sets, but the overall impact of differences in the imaging sequences acquired in the ex-vivo and in-vivo conditions remains a potentially significant confounding factor. Despite the printed scaffold, there was significant shrinkage of the LV cavity and tissue thickening in the healthy myocardium prior to the non-rigid registration step. We hypothesize that pressurizing the flexible 3D printed scaffold to reach LVEDP and the application of an excitation-contraction uncoupler [43] may further reduce the LV cavity reduction during ex-vivo imaging and represent a potentially valuable avenue for future work. There was no direct comparison between ex-vivo imaging acquired with and without the 3D printed scaffold, due to the time limitation during which ex-vivo imaging can be acquired, but the comparison with separately acquired ex-vivo imaging without the use of a scaffold indicates the improvement in the relationship between myocardial and LV cavity volumes resulting from the use of the scaffold. Finally, the process of segmentation and image registration uses three separate computational libraries, each chosen for the advantages offered by specific features within the library. While successful, this has resulted in a complex process of registration using multiple tools. This process could be simplified in future studies by development of a single interface with access to multiple libraries.

## Conclusions

The pattern of the morphological changes seen between the in-vivo and the ex-vivo LV differ between scar and healthy myocardium. A two-stage approach to optimize the co-registration of ex-vivo to in-vivo imaging to facilitate accurate comparison is reported. A 3D printed flexible scaffold based on the in-vivo shape of the LV cavity is an effective strategy to reduce morphological changes in the ex-vivo LV. This was combined with a non-rigid registration approach to reduce residual morphological changes, which further improved the co-registration and local comparison between in-vivo and ex-vivo imaging data.

## Supplementary information

**Additional file 1:** Supplementary data. **Figure S1.** Illustration of papillary muscle identification. Panel A: Short axis view of left ventricle at mid-cavity level. The papillary muscles are clearly separated from the left ventricular wall and were therefore not included in the LV segmentation at this level. Arrows indicate contrast between the papillary muscle body and the LV wall. Panel B: Short axis view on ex-vivo imaging at same level as panel A. In the ex-vivo condition the papillary muscles are pushed against the LV wall however a clear rim of contrast is visible between the papillary muscles and the LV wall so in the ex-vivo segmentation were not included in the LV wall at this level, reflecting the segmentation in the in-vivo imaging. Arrows indicate contrast between the papillary muscle body and the LV wall. Panel C: Short axis view of the LV below the level shown in panel A. At this level the papillary muscle is continuous with the LV wall and at this level was included in the LV segmentation. Panel D: Short axis view on ex-vivo imaging at the same level as panel C. There is no contrast between the bulge of the papillary muscles and the LV wall as they are continuous at this level, and were therefore included in the LV segmentation at reflecting the segmentation in the in-vivo imaging. Increased signal intensity around the endocardial surface in the ex-vivo condition may be contributed to by leaching of gadolinium into the saline adjacent the hearts were bathed in during ex-vivo imaging. **Figure S2.** in-vivo imaging. Panel A: Histogram of signal intensities within in-vivo segmented myocardium. Panel B: 3D volume rendering of segmented LV tissue (dark blue), aorta (gold) and scar (red) superimposed on SAX and LAX slices of in-vivo imaging. Panel C: Short axis slice of in-vivo imaging at mid-cavity level showing anteroseptal scar. Panel D: Corresponding slice of in-vivo imaging as panel C with segmentation of scar superimposed (red). **Figure S3.** Ex-vivo MR imaging and segmentation (imaging information outside of segmented LV myocardium including that from adherent pericardium removed for clarity). Panels A to C: Short axis (SAX) (A) and two long axis (LAX) (B / C) views of contrast enhanced ex-vivo imaging. Panels D to F: Same images as panels A to C with segmented scar (red) superimposed. Panel G: Signal intensity (SI) histogram within segmented myocardium. Red dashed-dotted line is mean SI within dense scar, black solid line is scar threshold, blue dashed line is mean SI within healthy tissue. Panel H: 3D volume rendering of segmented LV tissue (dark blue), aorta (gold) and scar (red) superimposed on SAX and LAX slices of ex-vivo imaging. **Figure S4.** Paired short axis slices from in-vivo (top) and ex-vivo (bottom) imaging using a rigid 3D printed scaffold. White arrow identifies rigid 3D printed scaffold, which generates a signal void in the imaging. Red arrow indicates the incision required for insertion of the rigid scaffold. Yellow arrow identifies scar in the in-vivo and ex-vivo imaging. **Figure S5.** Linear regression between volume of scar identified on in-vivo imaging using a full width at half maximum threshold and ex-vivo imaging. (DOCX 15892 kb)

## Data Availability

The datasets used and/or analyzed during the current study are available from the corresponding author on reasonable request.

## Abbreviations

AAR: Area at risk
ATP: Adeonsine Tri-phosphate
CMR: Cardiovascular magnetic resonance
FWHM: Full width half maximum
KCl: Potassium Chloride
LGE: Late gadolinium enhabncement
LV: Left ventricle
LVEDP: Left ventricular end-diastolic pressure
MI: Myocardial infarction
MPR: Multi-planar reconstruction
MV: Mitral valve
ROI: Region of interest
SI: Signal intensity
VT: Ventricular tachycardia
