# Supplementary material for: Improved co-registration of ex-vivo and in-vivo cardiovascular magnetic resonance images using heart-specific flexible 3D printed acrylic scaffold combined with non-rigid registration
Source: J Cardiovasc Magn Reson. 2019 Oct 10;21:62. doi: 10.1186/s12968-019-0574-z (PMC6785908; doi:10.1186/s12968-019-0574-z)
Supplement: Supplementary file 1 — Additional file 1: Supplementary data. Figure S1. Illustration of papillary muscle identification. Panel A: Short axis view of left ventricle at mid-cavity level. The papillary muscles are clearly separated from the left ventricular wall and were therefore not included in the LV segmentation at this level. Arrows indicate contrast between the papillary muscle body and the LV wall. Panel B: Short axis view on ex-vivo imaging at same level as panel A. In the ex-vivo condition the papillary muscles are pushed against the LV wall however a clear rim of contrast is visible between the papillary muscles and the LV wall so in the ex-vivo segmentation were not included in the LV wall at this level, reflecting the segmentation in the in-vivo imaging. Arrows indicate contrast between the papillary muscle body and the LV wall. Panel C: Short axis view of the LV below the level shown in panel A. At this level the papillary muscle is continuous with the LV wall and at this level was included in the LV segmentation. Panel D: Short axis view on ex-vivo imaging at the same level as panel C. There is no contrast between the bulge of the papillary muscles and the LV wall as they are continuous at this level, and were therefore included in the LV segmentation at reflecting the segmentation in the in-vivo imaging. Increased signal intensity around the endocardial surface in the ex-vivo condition may be contributed to by leaching of gadolinium into the saline adjacent the hearts were bathed in during ex-vivo imaging. Figure S2. in-vivo imaging. Panel A: Histogram of signal intensities within in-vivo segmented myocardium. Panel B: 3D volume rendering of segmented LV tissue (dark blue), aorta (gold) and scar (red) superimposed on SAX and LAX slices of in-vivo imaging. Panel C: Short axis slice of in-vivo imaging at mid-cavity level showing anteroseptal scar. Panel D: Corresponding slice of in-vivo imaging as panel C with segmentation of scar superimposed (red). Figure S3. Ex-vivo MR imagi [file 12968_2019_574_MOESM1_ESM.docx]

## Additional file 1

### CMR data acquisition - control

*In-vivo* CMR imaging: The protocol was approved by the Institutional Animal Care and Use Committee (IACUC) and conformed to the position of the American Heart Association on Research Animal Use and the Declaration of Helsinki. Imaging was performed using a 1.5 Tesla Philips Achieva (Philips Healthcare, Best, The Netherlands) scanner with a 32-channel cardiac phased array receiver coil. Scanning was performed after intravenous 0.2 mmol/L gadobenate dimeglumine (Multihance; Bracco Diagnostic Inc., Princeton, NJ) at 6 weeks following MI. Late gadolinium enhanced imaging was acquired using an isotropic navigator-gated ECG-triggered 3D inversion recovery sequence with a gradient echo readout (coronal orientation; linear k-space reordering; TE/TR/α= 2.7ms/6.1ms/25°; gating window= 7mm; voxel size= 1.2 x 1.2 x 1.2mm^3;^ Field of view (FOV)= 266 x 266 x 70 mm^3^, with full ventricular coverage).

#### *Ex-vivo* CMR imaging

In the *ex-vivo* imaging papillary muscles were included in the LV cavity segmentation if a clear rim of contrast was evident between the muscle body and the LV wall, in order to reflect as closely as possible the LV cavity segmentation approach taken with the *in-vivo* imaging (as shown in Figure S1).


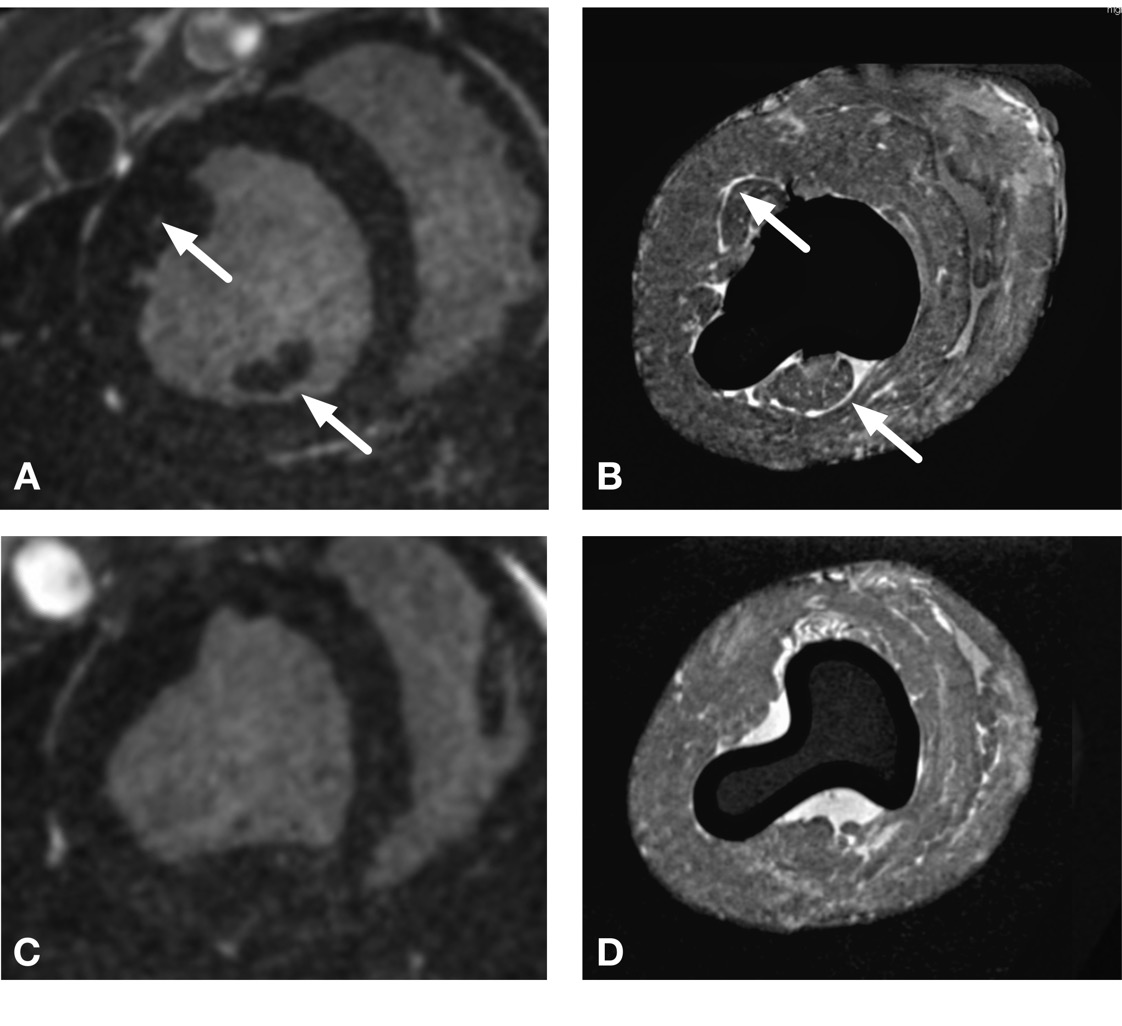


Figure S1: Illustration of papillary muscle identification. Panel A: Short axis view of left ventricle at mid-cavity level. The papillary muscles are clearly separated from the left ventricular wall and were therefore not included in the LV segmentation at this level. Arrows indicate contrast between the papillary muscle body and the LV wall. Panel B: Short axis view on *ex-vivo* imaging at same level as panel A. In the *ex-vivo* condition the papillary muscles are pushed against the LV wall however a clear rim of contrast is visible between the papillary muscles and the LV wall so in the *ex-vivo* segmentation were not included in the LV wall at this level, reflecting the segmentation in the *in-vivo* imaging. Arrows indicate contrast between the papillary muscle body and the LV wall. Panel C: Short axis view of the LV below the level shown in panel A. At this level the papillary muscle is continuous with the LV wall and at this level was included in the LV segmentation. Panel D: Short axis view on *ex-vivo* imaging at the same level as panel C. There is no contrast between the bulge of the papillary muscles and the LV wall as they are continuous at this level, and were therefore included in the LV segmentation at reflecting the segmentation in the *in-vivo* imaging. Increased signal intensity around the endocardial surface in the *ex-vivo* condition may be contributed to by leaching of gadolinium into the saline adjacent the hearts were bathed in during *ex-vivo* imaging.





Figure S2: *in-vivo* imaging. Panel A: Histogram of signal intensities within *in-vivo* segmented myocardium. Panel B: 3D volume rendering of segmented LV tissue (dark blue), aorta (gold) and scar (red) superimposed on SAX and LAX slices of *in-vivo* imaging. Panel C: Short axis slice of *in-vivo* imaging at mid-cavity level showing anteroseptal scar. Panel D: Corresponding slice of *in-vivo* imaging as panel C with segmentation of scar superimposed (red).


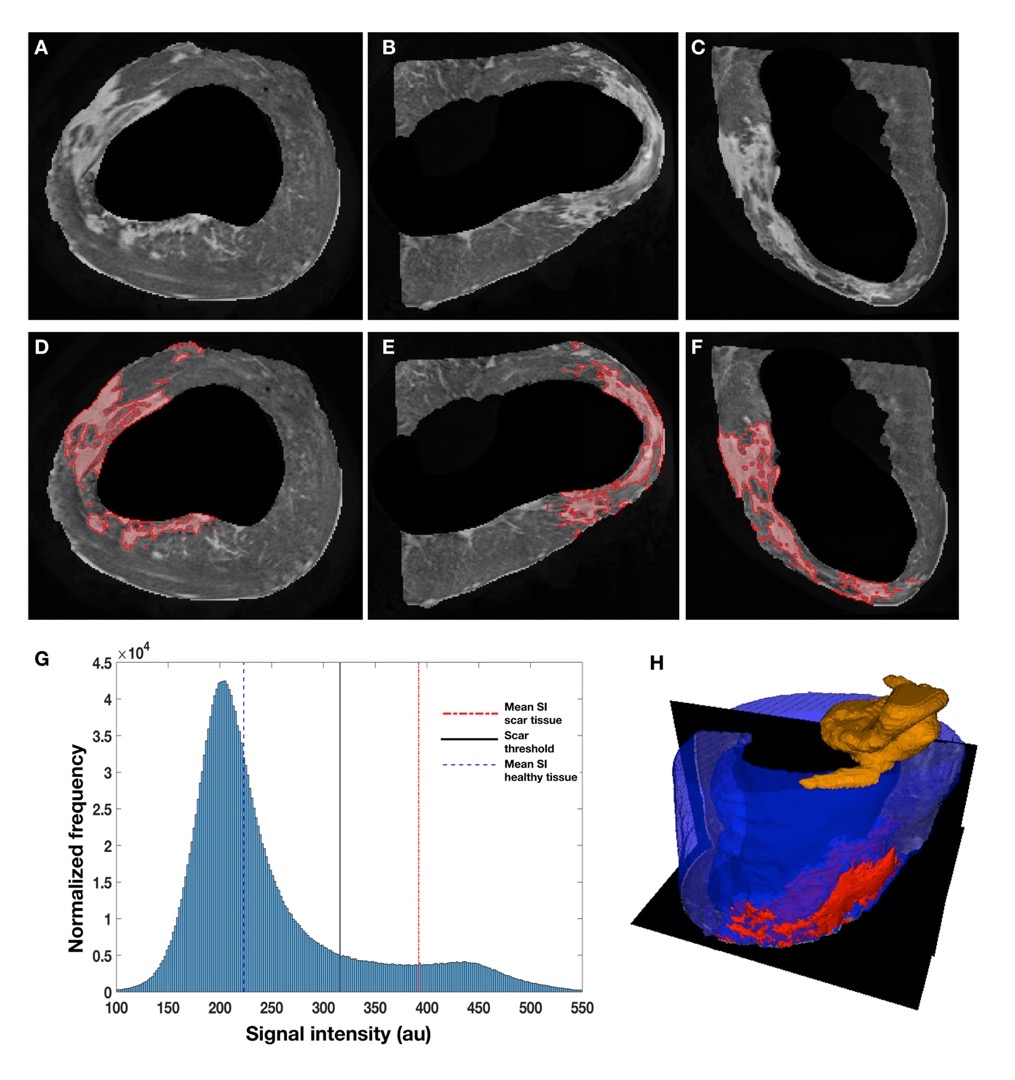


Figure S3: *Ex-vivo* MR imaging and segmentation (imaging information outside of segmented LV myocardium including that from adherent pericardium removed for clarity). Panels A to C: Short axis (SAX) (A) and two long axis (LAX) (B / C) views of contrast enhanced *ex-vivo* imaging. Panels D to F: Same images as panels A to C with segmented scar (red) superimposed. Panel G: Signal intensity (SI) histogram within segmented myocardium. Red dashed-dotted line is mean SI within dense scar, black solid line is scar threshold, blue dashed line is mean SI within healthy tissue. Panel H: 3D volume rendering of segmented LV tissue (dark blue), aorta (gold) and scar (red) superimposed on SAX and LAX slices of *ex-vivo* imaging.


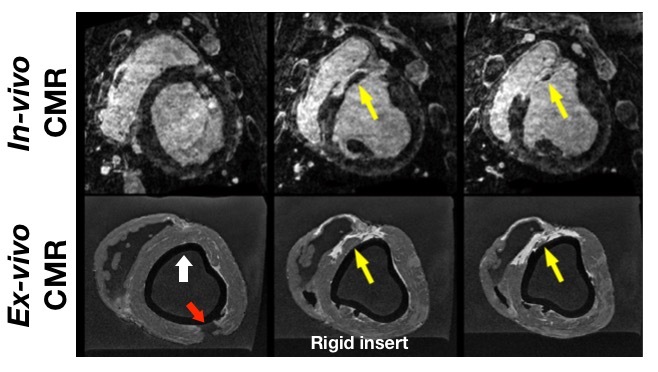


Figure S4: Paired short axis slices from *in-vivo* (top) and *ex-vivo* (bottom) imaging using a rigid 3D printed scaffold. White arrow identifies rigid 3D printed scaffold, which generates a signal void in the imaging. Red arrow indicates the incision required for insertion of the rigid scaffold. Yellow arrow identifies scar in the *in-vivo* and *ex-vivo* imaging.

**Results**

*
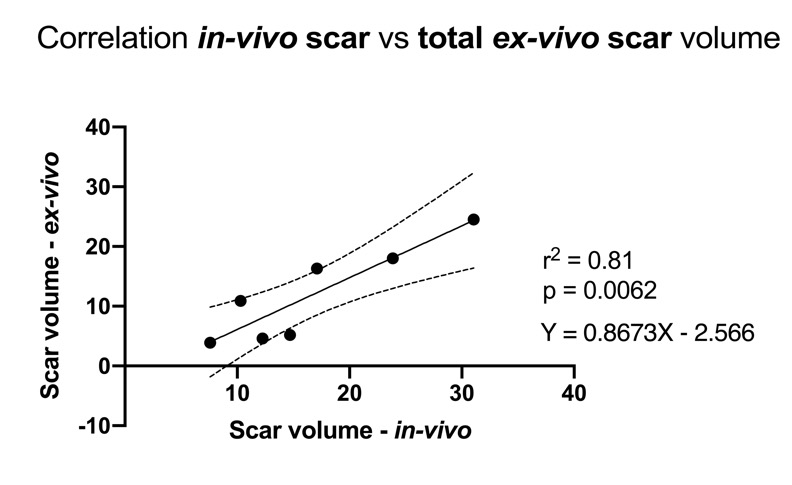
*

Figure S5 Linear regression between volume of scar identified on in-vivo imaging using a full width at half maximum threshold and ex-vivo imaging.
